# Microstructure Evolution and Mechanical Stability of Retained Austenite in Thermomechanically Processed Medium-Mn Steel

**DOI:** 10.3390/ma12030501

**Published:** 2019-02-06

**Authors:** Adam Grajcar, Andrzej Kilarski, Aleksandra Kozłowska, Krzysztof Radwański

**Affiliations:** 1 Silesian University of Technology, Institute of Engineering Materials and Biomaterials, 18A Konarskiego Street, 44-100 Gliwice, Poland; aleksandra.kozlowska@polsl.pl; 2 Opel Manufacturing Poland Sp. Z o.o., 1 Adama Opla Street, 44-121 Gliwice, Poland; andrzej.kilarski@opel.com; 3 Institute for Ferrous Metallurgy, 12-14 K. Miarki Street, 44-100 Gliwice, Poland; kradwanski@imz.pl

**Keywords:** medium manganese steel, bainitic steel, transformation induced plasticity, retained austenite stability, interrupted tensile test, electron backscatter diffraction (EBSD) method

## Abstract

A microstructure evolution of the thermomechanically processed 3Mn-1.5Al type steel and mechanical stability of retained austenite were investigated during interrupted tensile tests. The microstructural details were revealed using scanning electron microscopy (SEM), electron backscatter diffraction (EBSD), and transmission electron microscopy (TEM) techniques. It was found that the strain-induced martensitic transformation began in central regions of the largest blocky-type grains of retained austenite and propagated to outer areas of the grains as the deformation level increased. At rupture, the mechanical stability showed only boundaries of fine blocky grains of γ phase and austenitic layers located between bainitic ferrite laths. The effects of various carbon enrichment, grain size, and location in the microstructure were considered. The martensitic transformation progress was the highest at the initial stage of deformation and gradually decreased as the deformation level increased.

## 1. Introduction

Growing requirements of the automotive industry in recent years have developed new steel grades characterized by a good combination of high strength, ductility, and reduced production costs [1,2]. Recently, a lot of studies were focused on the high manganese austenitic steels showing Transformation Induced Plasticity and Twinning Induced Plasticity (TRIP/TWIP) effects. These steels belong to the second generation of Advanced High Strength Steels (AHSS) due to their excellent mechanical properties [3]. The prime objective in the development of medium-Mn steels showing a TRIP effect (third generation of AHSS) is to minimize the cost issues and at the same time providing favorable mechanical properties. The multiphase medium-Mn steels are characterized by a microstructure consisting of ferrite and austenite or bainite/martensite and austenite. They can be produced as cold-rolled or thermomechanically processed (TMP) steel sheets. The cold-rolled steels require a heat treatment consisting of austenite + ferrite production by heating an initial martensitic microstructure to an intercritical region [4,5,6]. For the thermomechanically rolled steels, the multiphase microstructure can be obtained as a result of one-step or controlled cooling following hot-working or intermediate quenching [6,7,8,9,10,11]. Besides many advantages of medium Mn steels, some technological problems related to a plastic instability phenomenon (Portevin–Le Chatelier and Dynamic Strain Ageing effects) can occur [12,13].

One of the major challenges in the development of the medium-Mn steels is obtaining the desired amount of retained austenite with an optimal stability. The stability of γ phase depends on several factors. The material factors are related to a chemical composition of retained austenite, its morphology, grain size, and a type of surrounding phases [14,15,16,17]. Carbon strongly affects the austenite stabilization and decreases the *M*_s_ temperature. On one hand, the retained austenite characterized by a low carbon content can be easily transformed into martensite during plastic deformation, which results in reduced elongation. On the other hand, too high carbon content causes its excessive stabilization, which also causes a reduction in ductility [18]. Pereloma et al. [19] reported that the most favorable, gradual progress of the strain-induced transformation occurs when retained austenite contains 1.1%–1.8% C. An increased Mn amount leads to obtaining a high fraction of retained austenite (~10%–30%) [18,19]. At the same time, it was found [20,21,22,23] that the increased manganese concentration decreases the carbon enrichment of retained austenite. Sun et al. [24] reported that increasing the Mn amount from 7% to 10% resulted in the reduction of the mechanical stability of retained austenite due to the lower C concentration partitioned into the austenite during heat treatment.

The exterior factors affecting the mechanical stability of retained austenite include the deformation temperature, strain rate, and stress state. Increasing the deformation temperature results in some reduction of the mechanical properties of medium-Mn steels. It is due to the smaller contribution of the TRIP effect, i.e., the mechanical stability of retained austenite increases [25,26]. Strain rate and deformation temperature factors are connected because increasing the strain rate results in increasing the temperature, which makes the austenite more stable. On the other hand, the application of high strain rates results in increasing the number of deformation bands, which are potential martensite nucleation sites [27,28].

An analysis of the microstructure evolution as a function of increasing strain allows determining the contribution of the TRIP effect, which depends on the mechanical stability of retained austenite. For this purpose, the electron backscatter diffraction (EBSD) technique can be successfully applied. The EBSD method is useful for determining a volume fraction of microstructural constituents: bainite, ferrite, and austenite [29,30,31,32,33]. Petrov et al. [30] used this method to determine an amount of retained austenite in a TRIP-assisted steel deformed to 10%. The mechanical stability of γ phase was widely studied in case of the first generation AHSSs [34,35]. However, the medium-Mn steels were not well characterized yet, especially in case of thermomechanically processed sheets. Therefore, the aim of the present study is to determine the mechanical stability of retained austenite in the thermomechanically processed medium-Mn steel in a wide deformation level.

## 2. Experimentation

### 2.1. Material and Thermomechanical Processing

A thermomechanically processed medium-Mn steel with a chemical composition shown in Table 1 was studied in the work. The carbon content was limited to 0.17 wt.% to provide its weldability [36,37,38]. The addition of 3 wt.% Mn provided the stability of γ phase. To suppress carbide precipitation [39], the steel was alloyed by 1.7% aluminum [39]. Mo was added for solid solution strengthening and delaying ferrite formation.

**Figure 1:**
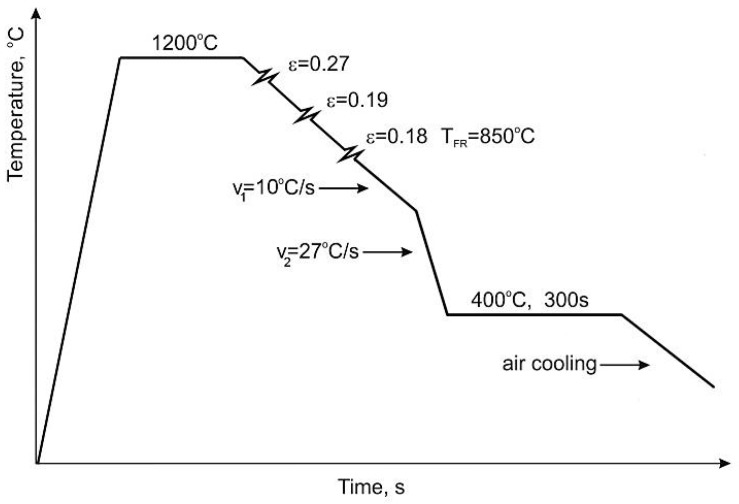
Parameters of thermomechanical processing of the investigated steel (*T*_FR_—temperature of finishing rolling).

The steel was cast using a Balzers VSG-50 vacuum induction furnace (Balzers, Asslar, Germany) under argon atmosphere. The steel samples were hot-forged in a temperature range 1200 to 900 °C. The flat samples were austenitized at 1200 °C for 3 h to minimize the segregation of alloying elements. A next step included hot-rolling of the samples in four passes to a thickness of 9 mm. These samples of 9 × 170 × 500 mm^3^ were used in further thermomechanical laboratory rolling. The final sheet thickness (4.5 mm) was obtained at 850 °C by the hot rolling in three passes, followed by the controlled cooling. Figure 1 shows the applied hot-rolling and cooling conditions. The detailed information on the thermomechanical treatment conditions was listed in Table 2. The steel sheet samples after the thermomechanical rolling were slowly cooled (~10 °C/s) to 700 °C. In the next step, they were cooled (~27 °C/s) to an isothermal holding temperature of 400 °C and annealed at this temperature for 300 s. Finally, the sheet specimens were air cooled to room temperature.

### 2.2. Tensile Tests

**Figure 2:**
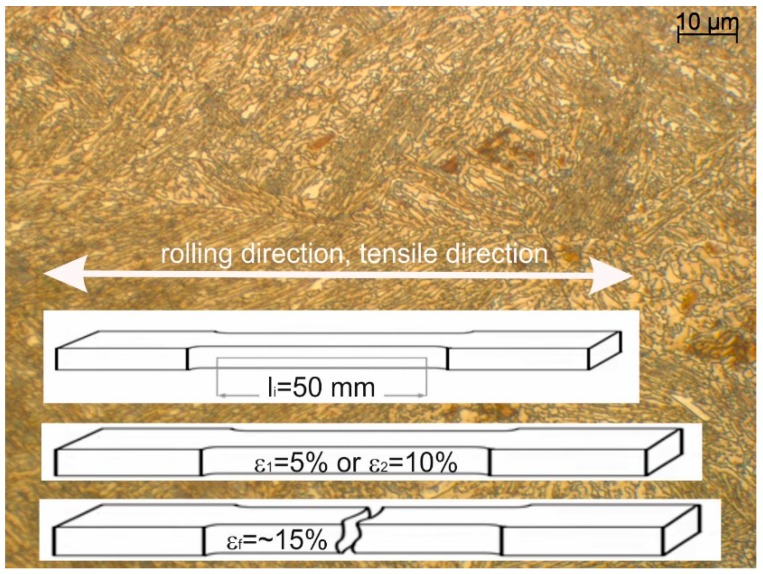
The macroscopic shape of samples used for interrupted tensile tests in respect of the microstructure and rolling and tensile directions.

Tensile test samples were prepared from 4.5 mm thick sheets according to the direction of hot rolling. The gauge length (*l*_i_) and width of 50 and 12.5 mm were used for the static tensile tests. Figure 2 shows the schematic of the macroscopic shape of samples used for interrupted tensile tests in respect of the microstructure and rolling and tensile directions. The uniaxial tensile tests were interrupted at defined strain values of 5% (ε_1_), 10% (ε_2_), and at the final rupture (ε_f_–15%) using the standard tensile test machine (Zwick Z/100, Zwick/Roell, Ulm, Germany). The tensile tests were performed to monitor the microstructure evolution at the different strain levels corresponding to a different progress of strain-induced martensitic transformation of the retained austenite.

### 2.3. Microstructural Characterization

Microstructural characterization was performed using the undeformed specimens (initial state) and the longitudinal samples deformed to the elongation 5%, 10%, and up to rupture. Microstructural observations were carried out using scanning electron microscopy (SEM), transmission electron microscopy (TEM) and electron backscatter diffraction (EBSD) methods. Changes in the amount of retained austenite at the different elongation levels were determined using the EBSD method.

Samples for the SEM were prepared according to the direction of hot rolling (initial state) and along the direction of tensile load. Specimens were mechanically ground with SiC paper up to 1500 grid, then polished with a diamond paste and finally etched in 5% nital to reveal the microstructure. The microstructures were examined by scanning electron microscope (Zeiss SUPRA 25, Carl Zeiss AG, Jena, Germany). The energy used for the analysis was 20 kV. The backscattered electron (BSE) detection mode was applied. TEM lamellas obtained by Focused Ion Beam (FIB) method (Quanta 3D 200i, FEI) were examined using a Titan 80-300, FEI transmission electron microscope (FEI, Hillsboro, OR, USA).

In order to determine the amount of retained austenite, the EBSD method was applied. An amount of γ phase was determined based on the average values of three measurements. The investigations were carried out by means of a high-resolution scanning electron microscope FEI Inspect F (FEI, Hillsboro, OR, USA). Samples for the EBSD examination were prepared by mechanical grinding and polishing following the classical procedure with a final polishing step of 0.1 µm Al_2_O_3_ paste. The last sample preparation step was electrolytic polishing using a TenuPol-5 device (Struers, Ballerup, Denmark) and A8 electrolyte by Struers, working at voltage 58 V during 40 s. The investigations were performed at a sample tilt angle of 70° towards normal to an electrooptic beam, using the acceleration voltage of 20 kV. Scanning was carried out at a scan step from 0.02 to 0.04 µm. The EBSD data were post processed using the TSL® OIM (Analysis 5) software. The grain size data were obtained using a grain tolerance angle of 5% and the minimum grain size was 2 pixels. These parameters are commonly used in this type of analysis [29,30]. The CI parameter (confidence index) was determined for each analysis. Data points characterized by the CI lower than 0.05 were not taken into account during the analysis. The following maps were analyzed: IQ image quality maps, phase distribution maps, crystallographic orientation distribution (inverse pole figure), and misorientation angle maps. The image quality maps were used to distinguish microstructural constituents based on Kikuchi patterns. Taking into account the similar IQ values for martensite and retained austenite [29], the γ phase was determined from the phase map, whereas the martensite amount was calculated as a remaining part.

## 3. Results and Discussion

### 3.1. Microstructure of Undeformed Samples 

**Figure 3:**
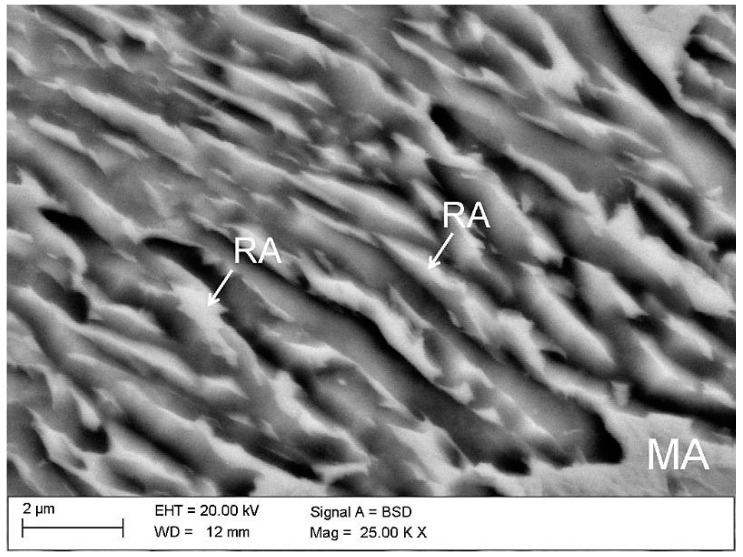
Microstructure of the specimen in the initial state (non-deformed) characterized by bainitic laths containing retained austenite and martensite–austenite (MA) constituents; retained austenite (RA).

The investigated steel is characterised by fine lath-type microstructure (Figure 3). Retained austenite is uniformly distributed in the bainitic matrix. No evidence of polygonal ferrite was observed due to the high steel hardenability. The SEM images revealed that only layered areas of γ phase located between the bainitic ferrite laths showed a thermal stability at room temperature. Blocky-type grains of retained austenite were partially transformed into martensite during the final cooling of the sheets to room temperature. This is due to a lower carbon content in larger grains, which resulted in a higher *M*_s_ temperature. This means that only some part of the austenite is thermally stable. Unfortunately, the transformed part does not contribute to the increase in plasticity as the TRIP effect during subsequent cold deformation [10,19]. As a consequence, a relatively high fraction of martensitic–austenitic (MA) constituents was already observed in the initial state.

**Figure 4:**
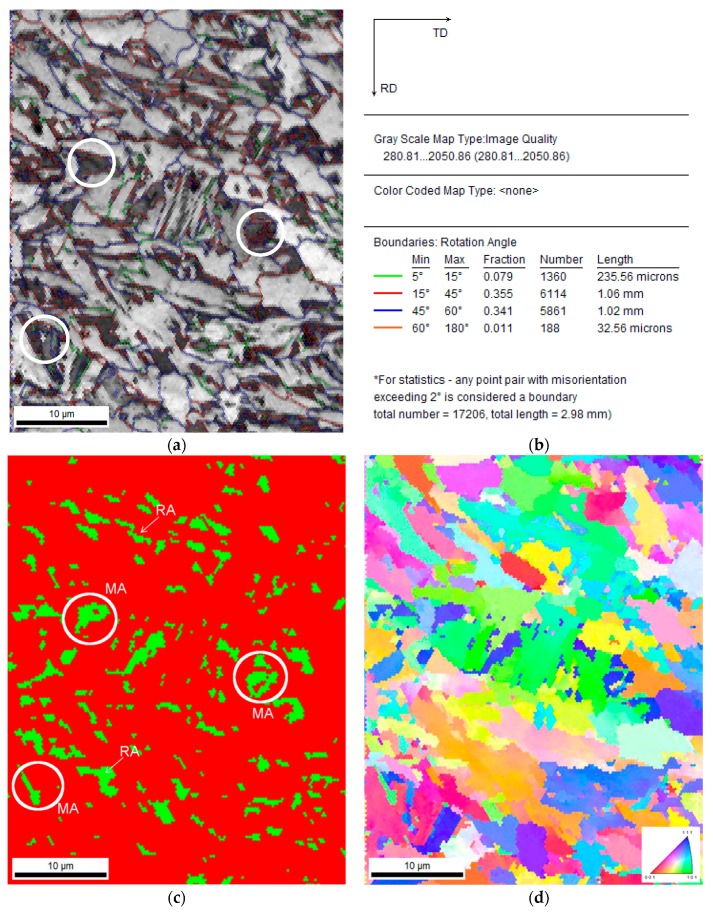
Image of the steel in the initial state (non-deformed). Image quality (IQ) map (**a**), statistical analysis of grain boundaries for the IQ map (**b**), phase distribution map—retained austenite (RA) as green (**c**), orientation map (**d**); MA (martensite–austenite) constituents.

The EBSD measurements led to determining the size, shape, and distribution of retained austenite. They allowed us also to establish the misorientation angles between individual grains. An IQ map presented in Figure 4 showed the differences between microstructural constituents. Retained austenite (RA) was identified on the basis of a crystal lattice, which was different from the matrix. In Figure 4a it corresponded approximately to dark areas. Based on the difference in gray levels of individual grains, the bainitic areas can be distinguished from the martensite. The darkest areas surrounded by austenite corresponded to the areas characterized by the worst diffraction quality (low IQ values), which can be identified as martensite. The latter phase is always characterized by the highest lattice deformation due to very high dislocation density. Hence, it shows the lowest IQ parameter [40,41].

The amount of low angle boundaries in the range of 5° to 15° was around 8% (Figure 4b), whereas the amount of high angle boundaries was estimated as 71%. The remaining part of the boundaries was characterized by a misorientation angle lower than 5°. According to the methodology proposed by Wu et al. [42], the boundaries located inside the grains correspond to the degree of advancement of the dislocation structure. The high density of boundaries characterized by the misorientation angle between 2° and 5° confirmed the high deformation level of the microstructure, associated with the relatively low temperature of finishing rolling, i.e., 850 °C. The total contribution of grain boundaries in the range 2° to 15° was ca. 29%. Based on the results of our previous research [43], it was found that reduction of finishing rolling temperature to 750 °C resulted in obtaining a higher amount of such grain boundaries (2° to 15°)—55%.

Figure 4c showed that the retained austenite was relatively uniformly distributed and significantly defragmented. This was reflected by a low sharpness of the Kikuchi lines in the martensitic and austenitic areas. The high-resolution ability of the EBSD method allowed us to identify the red areas located inside the blocky retained austenite grains (Figure 4c). Results of our research [43] confirmed that the central parts of the grains showed the smaller carbon enrichment. Hence, the martensitic transformation was initiated in these regions already upon cooling. Based on the crystallographic orientation map (Figure 4d), one can see that the grains exhibited a random crystallographic orientation.

**Figure 5:**
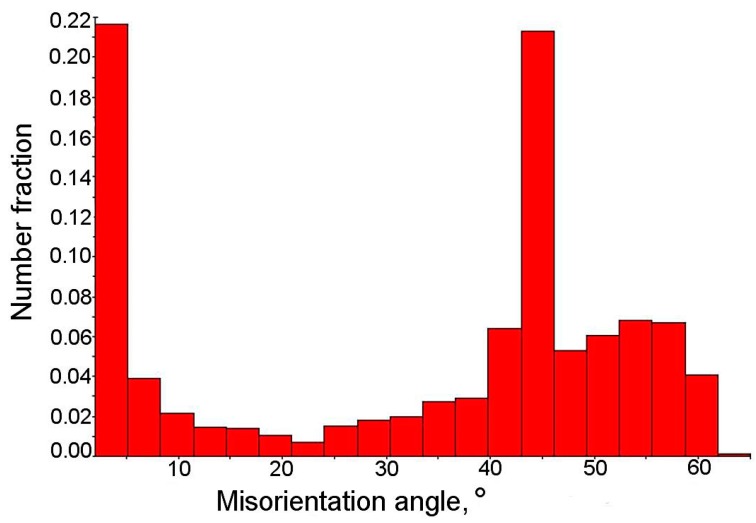
Distribution of misorientation angles in the initial state.

From the misorientation angle distribution (Figure 5), it was evident that a significant fraction of grain boundaries showed the misorientation angle close to 45°. This indicated the possibility of special crystallographic relations between the γ phase and bainite [43]. The γ and α phases are characterized by the special crystallographic orientation relationships, which are based on the correspondence of certain crystallographic planes and directions in the FCC (Face Centered Cubic) and the BCC (Body Centered Cubic) lattices [30]. It is well known that between the retained austenite and bainitic ferrite the Kurdjumov–Sachs (K-S) and Nishiyama–Wasserman (N-W) relationships occur. It means that some characteristic planes and parallel directions can be distinguished. Moreover, each orientation can be expressed by rotating the site around a parallel direction to the <uvw> (crystal direction) direction in one of the crystals by a certain angle, which leads to a superposition of the coordinate systems of both crystals [44]. These relationships were previously confirmed by Zaefferer et al. [44] in 0.2C-1.4Mn-0.5Si-07Al steel, and also by Wasilkowska et al. [40] in 0.2C-1.5Mn-1.5Si steel.

### 3.2. Microstructure Evolution during Interrupted Tensile Test

**Figure 6:**
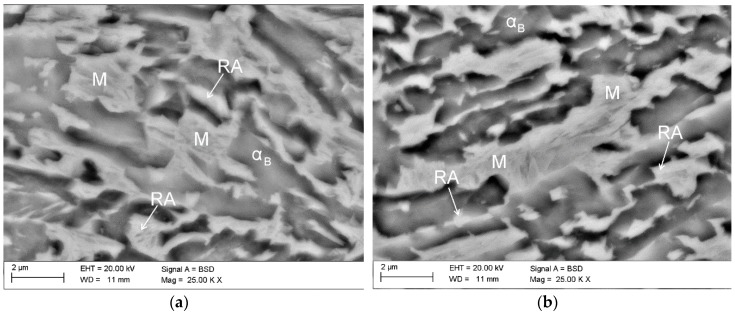

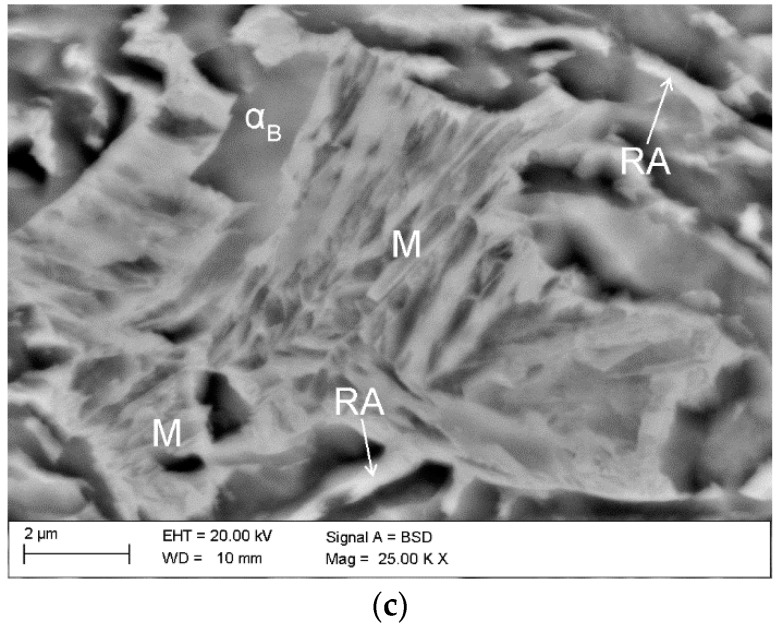
Microstructures showing the evolution of strain-induced martensitic transformation of retained austenite (RA) obtained in interrupted tensile tests. Specimens deformed to elongation values: 5% (**a**), 10% (**b**), and up to rupture (**c**); M—martensite, α_B_—bainitic ferrite.

A detailed analysis of the microstructure evolution during cold tensile straining allowed us to characterize the kinetics of TRIP effect in the investigated steel. The microstructure of the investigated steel deformed by a static tensile test to the elongation value 5% was shown in Figure 6a. One can see that at the initial stage of deformation, the strain-induced martensitic transformation was present in almost all blocky austenite grains larger than ~1 μm. For most of grains, the transformation occurred in the central part of the grain, while the outer areas of the grain remained untransformed. This confirmed that boundaries of γ phase grains located closely to bainitic ferrite were more enriched in carbon, whereas the central parts of the grains possessed smaller carbon content due to the smaller carbon diffusion efficiency [43]. The carbon profiles show the highest peaks at the ferrite/austenite boundary and they decrease towards a central part of the grain [6,9]. There is no Mn enrichment in the austenitic phase because it would require much higher temperatures and times [4]. This is why the reduction of the overall grain size is so important microstructural factor for a gradual (optimal) progress of strain-induced martensitic transformation. The obtained martensite was characterized by lath-type morphology. The formed martensite laths divided retained austenite grains into smaller blocks, which may contribute to the further increase of its mechanical stability [15,45]. Moreover, the hydrostatic pressure introduced by the newly formed martensitic plates favored the mechanical stabilization of untransformed austenite [34,46,47].

Increasing the deformation level to 10% resulted in the orientation of austenite grains according to the tensile stretching direction (Figure 6b). In this case, the martensitic transformation proceeded also in smaller austenitic grains, elongated according to the applied load. However, the boundaries of γ phase and thin layers remained untransformed. A further increase of the deformation level to rupture (~15%) resulted in the martensitic transformation of almost all blocky austenite grains. The transformation was also propagated to grain boundaries. After the rupture, only thin layers of γ phase with a thickness below ~0.3 μm remained stable (Figure 4c).

**Figure 7:**
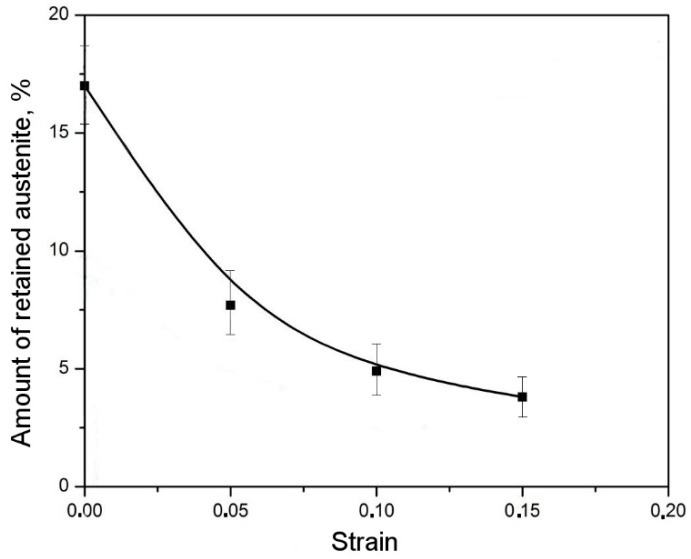
Change of retained austenite fraction as a function of strain level.

The change in the amount of retained austenite as a function of strain was presented in Table 3. In the deformation range from 0 to 0.05, about 50% of the total amount of retained austenite (detected at the initial state) transformed into martensite (Table 3). For the higher deformation levels, a fraction of the initial austenite transformed into martensite was lower. For different TRIP steels with a ferritic matrix, the highest increase in the martensite fraction also occurred at the initial deformation stage [40,41]. The amount of transformed grains corresponded roughly to the blocky grains. As the deformation level increases, the amount of blocky austenitic grains becomes smaller. The transformation was spread to the boundaries of blocky grains areas and occurred in layers of larger thickness. The remaining layers of retained austenite were affected by the hydrostatic pressure exerted by bainitic laths. Hence, they showed less tendency to be transformed. Therefore, in the deformation range of 0.05 to 0.1, an increase in the amount of martensite was significantly lower when compared to the initial strain level (Figure 7). The total elongation value of the investigated steel was ca. 15% [22]. An amount of the retained austenite stable under rupture conditions was determined as 4.8%. This means that near 70% of the initial γ phase was transformed into martensite. The calculated amount of the strain-induced martensite formed at particular strain levels is listed in Table 3.

The retained austenite fraction measured from the EBSD maps was compared to the volume fractions measured using X-ray diffraction (Table 3). The results are in good agreement. Less retained austenite in the EBSD method is due to a limited resolution of the analysis. Hence, the smallest blocky retained austenite grains and thin layers of this phase cannot be revealed [29,30,31]. These microstructural components can be detected using the X-ray diffraction [15,25].

**Figure 8:**
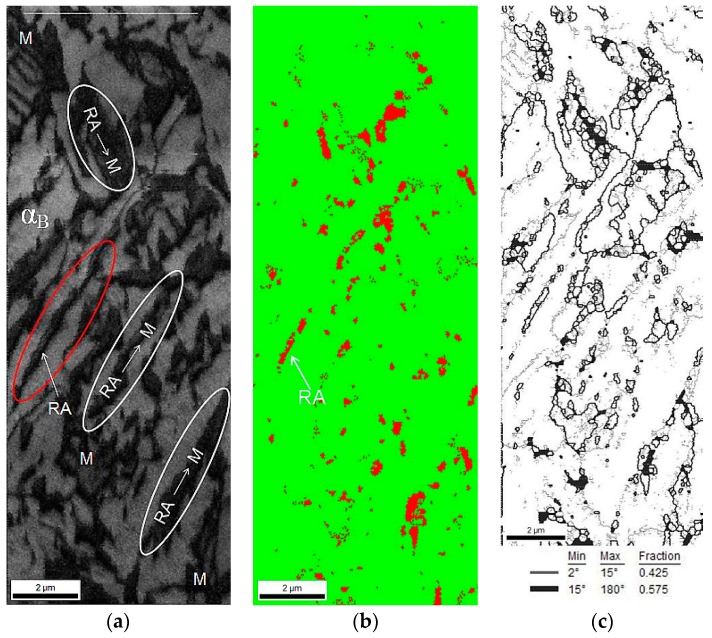

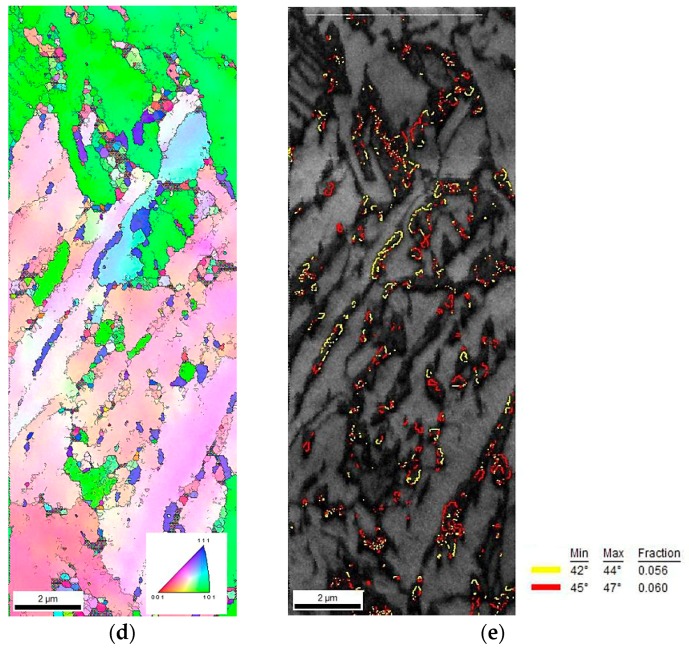
Image of the 3Mn-1.5Al steel deformed to 5% strain; IQ map (**a**), phase distribution map (retained austenite as red) (**b**), low-angle (2°–15°—thin line) and high-angle (15°–180°—thick line) boundaries map (**c**), inverse pole figure (**d**), grain boundaries of austenite and martensite/bainitic ferrite showing the Kurdjumov–Sachs (yellow) and Nishiyama–Wasserman (red) crystallographic relationships (**e**).

A phase identification was carried out using the EBSD method. The amounts of retained austenite and strain-induced martensite were determined for specimens at different deformation levels. The maps presented in Figure 8 showed the morphological details of the specimen elongated to 5%. Due to the high defects density related to the cold deformation, a lot of areas observed in Figure 8a were dark. The brighter color corresponded to the bainitic ferrite laths and the darker areas were identified as MA type constituents. Based on the phase distribution map (Figure 8b), the martensitic regions can be identified due to the fact that the martensite always has to be located at austenitic areas. Thus, the darkest areas observed in Figure 8b were identified as martensite. In case of blocky grains of γ phase transformed into martensite, small amount of the austenite was detected around the formed strain-induced martensite. For lath-type regions, it was observed that only some part of austenite transformed into martensite. It was due to the higher mechanical stability of austenitic films located between the bainitic ferrite laths. Figure 8c showed a significant increase in the amount of low angle boundaries in the range from 2° to 15°. Their amount rose from 29% detected at the initial state, to ca. 42% for the specimen elongated to 5% (Figure 4a,b). This indicated a significant increase in the dislocation density during plastic deformation. A detailed data on the percentage amount of the low-angle boundaries and high-angle boundaries can be found in Table 3. The progressive evolution of the increase in dislocation density as a function of strain is indirectly manifested by the growing amount of the low-angle boundaries.

A map presented in Figure 8d indicated that the majority of retained austenite grains had a preferential crystallographic orientation in the <111> direction. Based on the data presented in Figure 8, one can conclude that a large number of grains exhibited a crystallographic misorientation angle close to 45°, similar to the specimen at the initial state (Figure 5). The advantage of the EBSD method (related to its resolution) is the ability to distinguish the misorientation angle value to an accuracy of about 1° [48]. In Figure 8e, the high angle boundaries in a range of 42° to 44° (yellow) and in a range from 45° to 47° (red) were marked. These boundaries corresponded to the Kurdjumov–Sachs (K-S) and Nishiyama–Wasserman (N-W) orientation relationships between retained austenite and martensite.

**Figure 9:**
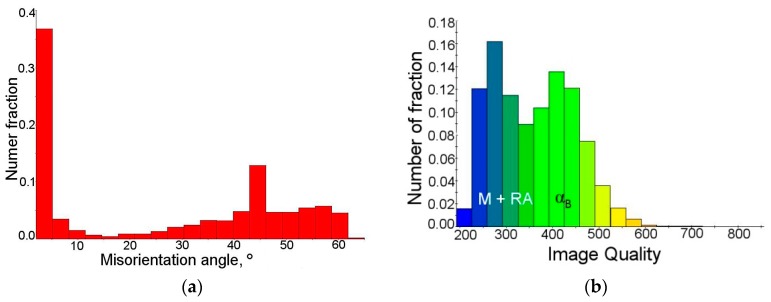
Distribution of misorientation angle (**a**) and IQ parameter (**b**) of the sample elongated to 5%.

Figure 9a confirmed that cold deformation affected significantly the increase in the amount of grain boundaries characterized by misorientation angles less than 5°. A fraction of grain boundaries showing the Kurdjumov–Sachs (K-S) and Nishiyama–Wasserman (N-W) relationships was estimated as 14%, whereas the amount of this kind of grain boundaries at the initial state was ca. 22%. As the deformation level increased the amount of retained austenite decreased. Thus, a fraction of γ phase showing crystallographic relationships with the bainitic ferrite became lower. On the other hand, new preferential interphase boundaries between the remaining austenite and the newly formed martensite were formed. Figure 9b showed the frequency of grains with a defined image quality. Two peaks indicating the occurrence of structural constituents characterized by various deflections of the crystal lattice can be identified. This graph determined the quality of the obtained diffractions in the form of the Kikuchi lines. Based on the quality map (Figure 8a), one can conclude that first peak of low image quality corresponded to the retained austenite and martensite (M + RA) regions, whereas the second, wider one corresponded to the bainitic ferrite regions (Figure 9b).

**Figure 10:**
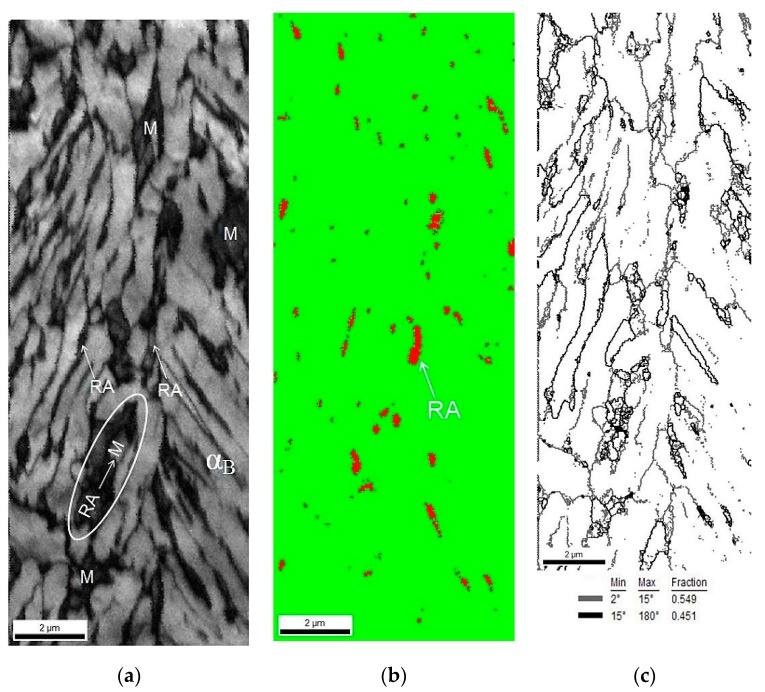

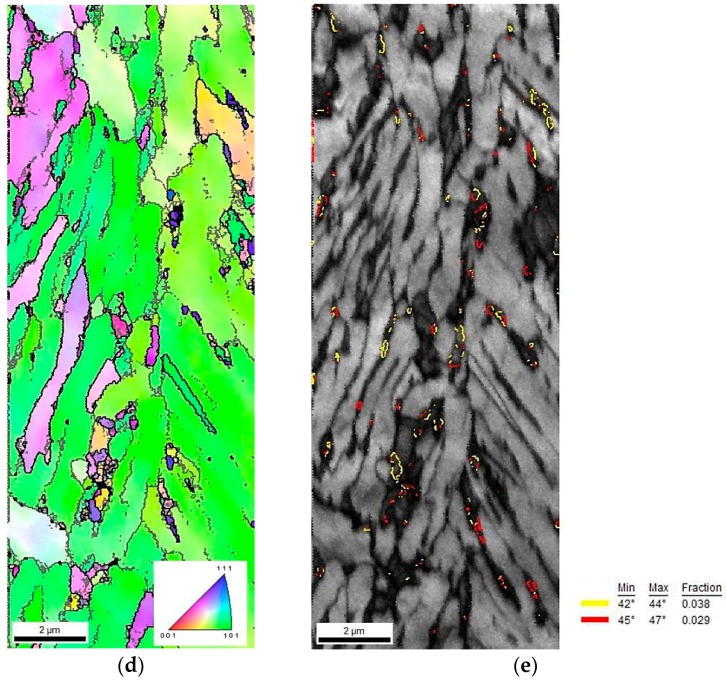
Image of the 3Mn-1.5Al steel deformed to 10% strain; IQ map (**a**), phase distribution map (retained austenite as red) (**b**), low-angle (2°–15°—thin line) and high-angle (15°–180°—thick line) boundaries map (**c**), inverse pole figure (**d**), grain boundaries of austenite and martensite/bainitic ferrite showing the Kurdjumov–Sachs (yellow) and Nishiyama–Wasserman (red) crystallographic relationships (**e**).

Increasing the deformation level to 10% caused the transformation of almost all blocky austenite grains into martensite (Figure 10a). Only layers of various thicknesses located between the bainitic ferrite remained stable (Figure 10b). The amount of low angle boundaries in a range of 2° to 15° was ca. 55% (Figure 10c). This showed that for a strain range 5% to 10% an increase of the low angle boundaries fraction was ca. 12.5% when compared to the results presented in Figure 8c. It proved the intense development of dislocation substructure. These boundaries formed usually inside or around the austenitic–martensitic areas. Retained austenite still showed the crystallographic orientation close to <111> direction (Figure 10d). A total amount of the high angle boundaries corresponding to the Kurdjumow–Sachs (yellow) and Nishiyama–Wasserman (red) relationships decreased from 11.6% (Figure 8e) to 6.7% (Figure 10e). This was due to a gradual decrease in the amount of retained austenite. It can be seen that for a deformation level of 10%, a fraction of boundaries corresponding to the K-S relationship was greater than showing the N-W orientation. For a deformation level of 5%, this tendency was reversed (Figure 10e).

**Figure 11:**
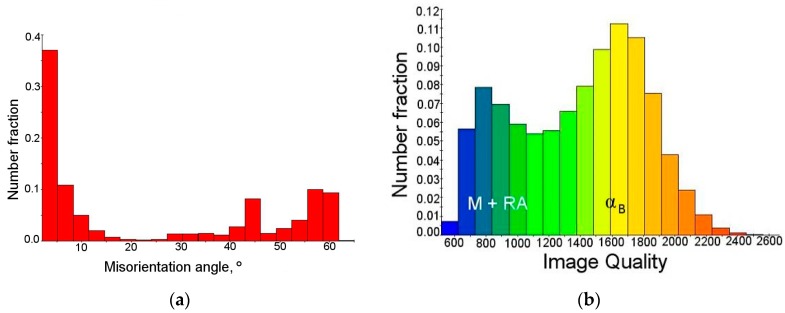
Distribution of misorientation angle (**a**) and IQ parameter (**b**) of the sample elongated to 10%.

Figure 11a showed an increase in the amount of low-angle boundaries and a decrease in the fraction of grains characterized by the misorientation angle close to 45°. Based on the chart showing the distribution of the diffraction quality of individual grains (Figure 11b), it can be concluded that, in comparison to the sample deformed to 5%, the range of grains exhibiting a various image quality was higher. It corresponded to a different deformation degree of individual structural constituents.

**Figure 12:**
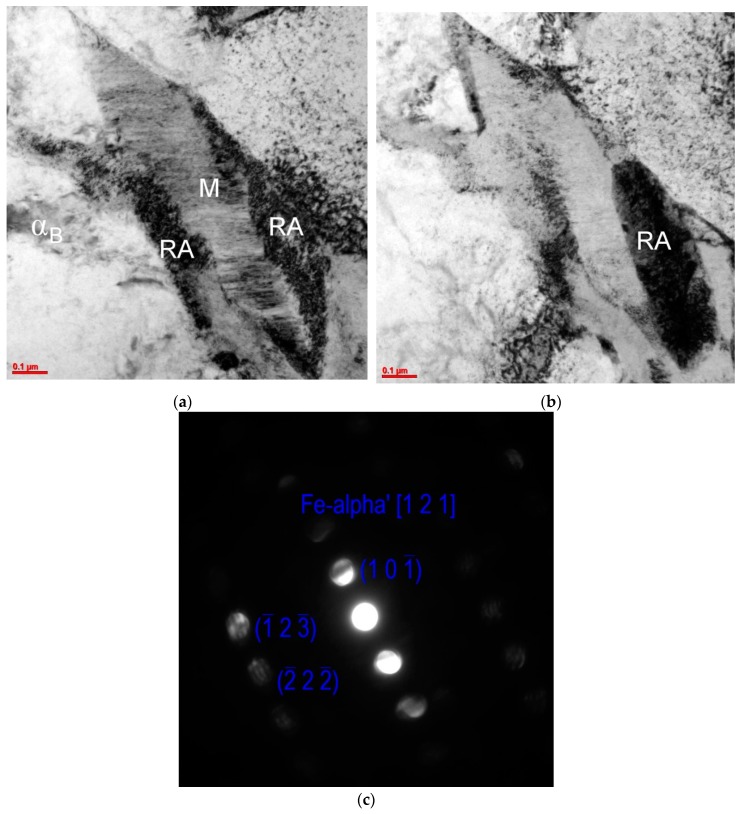
A middle part of the austenite layer transformed to fine-plate martensite in a sample deformed to 10%; a bright field with the contrast on martensite (M) (**a**), a bright field with a contrast on retained austenite (RA) (**b**), microdiffraction from the martensitic plates area (**c**).

The morphological details of strain-induced martensite obtained as a result of the applied tensile load were revealed by using TEM. Figure 12a showed the fine-plate martensite formed within the retained austenite layer (~0.4 μm thick) in the sample deformed to 10%. Moreover, it was confirmed that regions of the austenite located near a boundary with the bainitic ferrite remained mechanically stable (Figure 12b). It was evidence that the areas located near the bainitic ferrite were characterized by a higher concentration of carbon when compared to the areas located in the grain center [49,50].

## 4. Conclusions

Mechanical stability of retained austenite in thermomechanically rolled 3Mn-1.7Al steel is of major importance for its microstructure evolution during cold forming. The performed analyses enabled us to determine the effect of tensile strain level on the mechanical stability of retained austenite. Interrupted tensile tests were applied to monitor the martensite transformation progress. It was found that in the initial stage of deformation (5%) only central regions of the largest blocky-type grains of retained austenite were transformed. At the deformation level 5%, ca. 50% of the total amount of initial retained austenite transformed into martensite. Increasing the deformation level to 10% resulted in a reduction of martensitic transformation progress and its propagation to the boundary areas of blocky grains. Under rupture conditions thin layers of retained austenite and small defragmented grains remained only mechanically stable. The stabilization effect was caused by the reduction in a size of the austenite grains and the presence of the bainitic ferrite causing the hydrostatic pressure. This effect was supported by the compressive stresses accompanying the martensitic transformation. This promoted the stabilization of austenitic films. About 5% of the γ phase was detected after the rupture.

The bainitic ferrite and retained austenite as well as the strain-induced martensite and defragmented retained austenite showed the Kurdjumov–Sachs and Nishiyama–Wasserman relationships. The dislocation density increased with increasing deformation level, which was reflected in the increase of the number of low angle boundaries.

## Figures and Tables

**Table 1:** Chemical composition of the investigated steel, in wt.%.

Steel Type	C %	Mn %	Al %	Si %	Mo %	S %	P %	O %	N %
3Mn-1.5Al	0.17	3.3	1.7	0.22	0.23	0.014	0.010	0.0004	0.0043

**Table 2:** Conditions of the thermomechanical rolling.

Pass Number	Deformation Temperature (°C)	Sheet Thickness before a Pass (mm)	Sheet Thickness after a Pass (mm)	Absolute Reduction (mm)	Relative Strain (%)	Strain Rate (s^−1^)
1	1050	9.0	6.6	2.4	27	5
2	950	6.6	5.4	1.2	19	7
3	850	5.4	4.5	0.9	18	8

**Table 3:** Amount of retained austenite at different deformation levels determined using the EBSD and XRD methods.

**Deformation Level**	ε_0_ = 0%	ε_1_ = 5%	ε_2_ = 10%	ε_f_ ~ 15% (Rupture)
**Amount of Retained Austenite, %** **(EBSD Method)**	16.8 ± 1.8	8.3 ± 1.3	5.7 ± 1.2	4.8 ± 1.0
**Amount of Retained Austenite, %** **(XRD Method)**	17.3	8.6	6.5	5.4
**Fraction of Low-Angle Boundaries, %**	7.9	7.5	20.2	27.5
**Fraction of High-Angle Boundaries, %**	70.7	57.5	45.1	39.3
**Calculated Amount of the Initial Retained Austenite Transformed into Martensite, %**	-	51	66	71
